# 1,1,1-Trifluoro-4-(thio­phen-2-yl)-4-[(2-{[4,4,4-trifluoro-3-oxo-1-(thio­phen-2-yl)but-1-en-1-yl]amino}­eth­yl)amino]­but-3-en-2-one

**DOI:** 10.1107/S1600536811036889

**Published:** 2011-09-17

**Authors:** Abdullah M. Asiri, Abdulrahman O. Al-Youbi, Hassan M. Faidallah, Seik Weng Ng

**Affiliations:** a Chemistry Department, Faculty of Science, King Abdulaziz University, PO Box 80203 Jeddah, Saudi Arabia; b Center of Excellence for Advanced Materials Research, King Abdulaziz University, PO Box 80203 Jeddah, Saudi Arabia; c Department of Chemistry, University of Malaya, 50603 Kuala Lumpur, Malaysia

## Abstract

The asymmetric unit of the diamine compound, C_18_H_14_F_3_N_2_O_2_S_2_, consists of two mol­ecules; the C=C double bond has a *Z* configuration in the C_4_H_3_S—C=C—C(=O)—C segment. The –NH—CH_2_—CH_2_—NH chain adopts a twisted U-shape. The amino group is an intra­molecular hydrogen-bond donor to the carbonyl group; the intra­molecular hydrogen bond generates a six-membered ring. In both mol­ecules, the thienyl rings are disordered over two positions; the occupancies of the major components are 0.817 (4) and 0.778 (4) in one mol­ecule and 0.960 (4) and 0.665 (4) in the other. One of the trifluoro­methyl groups is disordered over two positions with the major component having 0.637 (8) occupancy.

## Related literature

For the synthesis, see: Wang & Tong (1995 ▶). For related structures, see: Bresciani-Pahor *et al.* (1979 ▶); Haider *et al.* (1981 ▶).
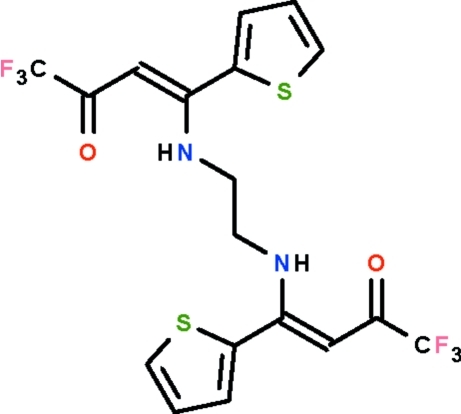


## Experimental

### 

#### Crystal data

C_18_H_14_F_6_N_2_O_2_S_2_
*M* *_r_* = 468.43
Orthorhombic, 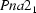

*a* = 20.4520 (4) Å
*b* = 12.5201 (2) Å
*c* = 15.8328 (2) Å
*V* = 4054.16 (11) Å^3^
*Z* = 8
Mo *K*α radiation
μ = 0.33 mm^−1^
*T* = 100 K
0.30 × 0.25 × 0.20 mm

#### Data collection

Agilent SuperNova Dual diffractometer with an Atlas detector
Absorption correction: multi-scan (*CrysAlis PRO*; Agilent, 2010 ▶) *T*_min_ = 0.906, *T*_max_ = 0.936
40183 measured reflections
9198 independent reflections
8265 reflections with *I* > 2σ(*I*)
*R*_int_ = 0.037

#### Refinement

*R*[*F* ^2^ > 2σ(*F* ^2^)] = 0.046
*wR*(*F* ^2^) = 0.122
*S* = 1.05
9198 reflections
633 parameters
242 restraints
H atoms treated by a mixture of independent and constrained refinement
Δρ_max_ = 0.61 e Å^−3^
Δρ_min_ = −0.59 e Å^−3^
Absolute structure: Flack (1983 ▶), 4340 Friedel pairs
Flack parameter: 0.01 (7)

### 

Data collection: *CrysAlis PRO* (Agilent, 2010 ▶); cell refinement: *CrysAlis PRO*; data reduction: *CrysAlis PRO*; program(s) used to solve structure: *SHELXS97* (Sheldrick, 2008 ▶); program(s) used to refine structure: *SHELXL97* (Sheldrick, 2008 ▶); molecular graphics: *X-SEED* (Barbour, 2001 ▶); software used to prepare material for publication: *publCIF* (Westrip, 2010 ▶).

## Supplementary Material

Crystal structure: contains datablock(s) global, I. DOI: 10.1107/S1600536811036889/xu5326sup1.cif

Structure factors: contains datablock(s) I. DOI: 10.1107/S1600536811036889/xu5326Isup2.hkl

Supplementary material file. DOI: 10.1107/S1600536811036889/xu5326Isup3.cml

Additional supplementary materials:  crystallographic information; 3D view; checkCIF report

## Figures and Tables

**Table 1:** Hydrogen-bond geometry (Å, °)

*D*—H⋯*A*	*D*—H	H⋯*A*	*D*⋯*A*	*D*—H⋯*A*
N1—H1n⋯O1	0.88 (1)	2.03 (3)	2.741 (3)	138 (3)
N2—H2n⋯O2	0.88 (1)	2.01 (3)	2.726 (3)	138 (3)
N3—H3n⋯O3	0.88 (1)	1.93 (3)	2.668 (3)	140 (3)
N4—H4n⋯O4	0.87 (1)	1.96 (3)	2.677 (3)	139 (3)

**Fig. 1.**
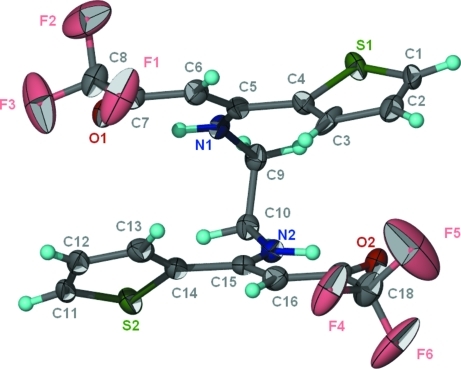
Thermal ellipsoid plot (Barbour, 2001) of one of the two independent molecules of C16H14F3N2O1S2 at the 70% probability level; hydrogen atoms are drawn as spheres of arbitrary radius. The disorder is not shown.

**Fig. 2.**
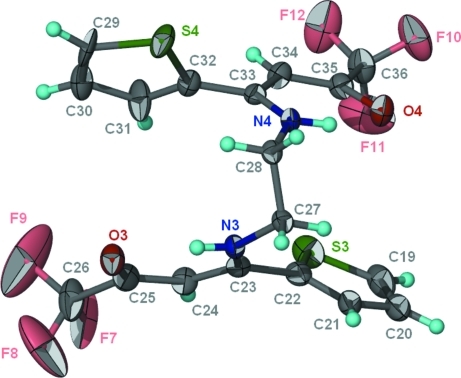
Thermal ellipsoid plot (Barbour, 2001) of second independent molecule of C16H14F3N2O1S2 at the 70% probability level; hydrogen atoms are drawn as spheres of arbitrary radius. The disorder is not shown.

## supplementary crystallographic information

### Comment

Ethylenediamine generally condenses with aldehydes and ketones to yield Schiff bases, which are typically yellow compounds whose coloration arises from the azomethine linkage. For some 1,3-diketones (such as acetylacetone and benzoylmethane), the diamine condenses with the carbony function of two ketone molecules. In the condensation product, the azomethine double-bond isomerizes to result in the formation of a secondary amine, as noted in the diamine, 4-((2-((1-methyl-3-oxo-but-1-enyl)amino)ethyl)amino)pent-3-en-2-one (from the condensation of ethylenediamine with acetylacetone) (Bresciani-Pahor *et al.*, 1979) and the analogous amine from the condensation of ethylenediamine with benzoyacetone (Haider *et al.*, 1981). The product from the condensation with 2-theonyltrifluoroacetone was first reported in 1995, but the authors of the study assigned it as (ethanediyldinitrilo)bis[fluoro(thienyl)butanone as they considered it a Schiff-base-alicyclic type of crown ether (Wang & Tong, 1995). The compound is, in fact, is diamine (Scheme I). The asymmetric unit of the diamine, C_16_H_14_F_3_N_2_O_1_S_2_, consists of two molecules; the C–C double-bond is of a *Z*-configuration. In the C_4_H_3_S–C═C–C(═O)–C segment, the thienyl ring and acetyl fragment are approximately coplanar. The amino group is an intramolecular hydrogen-bond donor to the carbonyl group, and the hydrogen bond generates a six-membered ring (Table 1). In both molecules, their thienyl rings are each disordered over two positions; one trifluoromethyl group is also disordered (Fig. 1 and Fig. 2). Whereas similar diamines are centrosymmetric molecules with the inversion center in the middle of the ethane link, the present compound is not. The –NH–CH_2_–CH_2_–NH chain adopts a twisted *U*-shape.

### Experimental

Ethylenediamine (0.6 g, 10 mmol) and the 2-theonyltrifluoroacetone (2.2 g, 10 mmol) in benzene (50 ml) were heated in a Dean-Stark trap until no more water was collected (in about 2 h). The solvent was removed and the residue was treated with a little methanol. The solid that separated was recystallized from ethanol to yield light yellow crystals.

### Refinement

Carbon- and nitrogen-bound H-atoms were placed in calculated positions [C–H 0.95–0.99 Å; *U*_iso_(H) 1.2*U*_eq_(C)] and were included in the refinement in the riding model approximation. The amino H-atoms were located in a difference Fourier map and were refined with a distance restraint of N–H 0.88±0.01 Å; their temperature factors were tied by a factor of 1.5.

All four thiophene rings are disordered over two positions in four of the five atoms; their *α*-carbon atoms are ordered. The formal carbon–carbon single-bond distances were restrained to 1.36 ± 0.01 Å and the double-bond distances to 1.42±0.01 Å wherease the carbon–sulfur distances bond distances were restrained to 1.71±0.01 Å. All 1,3-related sulfur–carbon distances were restrained to within 0.01 Å of each other. The thienyl rings are each restrained to lie on a plane. For the S1/C1/C2/C3/C4 and S1'/C2'/C3'/C4 disordered rings, the isotropic temperature factor of C3' was set to the equivalent anisotropic temperature factor of S1; the S1 atom was allowed to refine anisotropically. The isotropic temperature factors of the atoms of the minor components were similarly restrained to those the anisotropic temperature factors of the atoms of the major components.

One of the trifluoromethyl groups is disordered over two positions. All carbon–fluoroine distances were restrained to within 0.01 Å of each other. The six F-atoms were restrained to lie on a plane; their anisotropic temperature factors were tightly restrained to be nearly isotropic.emperature factors of the primed atoms were set to those of the umprimed ones.

Some atoms (F5, F7, C3 and C29) displayed somewhat elongated ellipsoids, which may be a consequence of the large number of restraints. For the disordered thienyl rings, the S1–C4 and C3–C4 bond distances are show differences in the Hirshfeld test.

Omitted from the refinement owing to bad disagreement was (1 1 -1).

### Crystal data

**Table:**

C_18_H_14_F_6_N_2_O_2_S_2_	*F*(000) = 1904
*M**_r_* = 468.43	*D*_x_ = 1.535 Mg m^−^^3^
Orthorhombic, *P**n**a*2_1_	Mo *K*α radiation, λ = 0.71073 Å
Hall symbol: P 2c -2n	Cell parameters from 16458 reflections
*a* = 20.4520 (4) Å	θ = 2.3–27.5°
*b* = 12.5201 (2) Å	µ = 0.33 mm^−^^1^
*c* = 15.8328 (2) Å	*T* = 100 K
*V* = 4054.16 (11) Å^3^	Block, light-yellow
*Z* = 8	0.30 × 0.25 × 0.20 mm

### Data collection

**Table:**

Agilent SuperNova Dual diffractometer with an Atlas detector	9198 independent reflections
Radiation source: SuperNova (Mo) X-ray Source	8265 reflections with *I* > 2σ(*I*)
Mirror	*R*_int_ = 0.037
Detector resolution: 10.4041 pixels mm^-1^	θ_max_ = 27.6°, θ_min_ = 2.3°
ω scans	*h* = −26→24
Absorption correction: multi-scan (*CrysAlis PRO*; Agilent, 2010)	*k* = −16→15
*T*_min_ = 0.906, *T*_max_ = 0.936	*l* = −20→20
40183 measured reflections	

### Refinement

**Table:**

Refinement on *F*^2^	Secondary atom site location: difference Fourier map
Least-squares matrix: full	Hydrogen site location: inferred from neighbouring sites
*R*[*F*^2^ > 2σ(*F*^2^)] = 0.046	H atoms treated by a mixture of independent and constrained refinement
*wR*(*F*^2^) = 0.122	*w* = 1/[σ^2^(*F*_o_^2^) + (0.0629*P*)^2^ + 2.3648*P*] where *P* = (*F*_o_^2^ + 2*F*_c_^2^)/3
*S* = 1.05	(Δ/σ)_max_ = 0.001
9198 reflections	Δρ_max_ = 0.61 e Å^−^^3^
633 parameters	Δρ_min_ = −0.59 e Å^−^^3^
242 restraints	Absolute structure: Flack (1983), 4340 Friedel pairs
Primary atom site location: structure-invariant direct methods	Flack parameter: 0.01 (7)

### Fractional atomic coordinates and isotropic or equivalent isotropic displacement parameters (Å^2^)

**Table:**

	*x*	*y*	*z*	*U*_iso_*/*U*_eq_	Occ. (<1)
S1	0.81914 (6)	0.35483 (9)	0.50113 (16)	0.0299 (3)	0.817 (4)
C3'	0.8183 (5)	0.3618 (12)	0.4772 (9)	0.030*	0.183 (4)
H3'	0.8582	0.4000	0.4718	0.036*	0.183 (4)
S2	0.77950 (7)	0.40257 (11)	0.05479 (16)	0.0309 (4)	0.778 (4)
C13'	0.7645 (6)	0.4026 (14)	0.0719 (9)	0.031*	0.222 (4)
H13'	0.8073	0.3795	0.0575	0.037*	0.222 (4)
S3	0.52193 (5)	0.51039 (8)	0.63447 (16)	0.0349 (3)	0.960 (4)
C21'	0.5388 (9)	0.502 (3)	0.617 (4)	0.035*	0.040 (4)
H21'	0.4987	0.4743	0.6380	0.042*	0.040 (4)
S4	0.63026 (8)	0.23117 (13)	0.98065 (17)	0.0337 (4)	0.665 (4)
C31'	0.6224 (5)	0.2405 (11)	0.9654 (7)	0.034*	0.335 (4)
H31'	0.6605	0.2696	0.9909	0.040*	0.335 (4)
C1	0.7728 (2)	0.2583 (3)	0.5476 (3)	0.0292 (10)	0.817 (4)
H1	0.7856	0.2198	0.5966	0.035*	0.817 (4)
C2'	0.8064 (7)	0.2803 (13)	0.5393 (9)	0.029*	0.183 (4)
H2'	0.8371	0.2574	0.5805	0.035*	0.183 (4)
C2	0.71492 (19)	0.2427 (3)	0.5062 (3)	0.0273 (9)	0.817 (4)
H2	0.6831	0.1907	0.5210	0.033*	0.817 (4)
C1'	0.7452 (8)	0.2410 (12)	0.5305 (9)	0.027*	0.183 (4)
H1'	0.7276	0.1863	0.5653	0.033*	0.183 (4)
C3	0.7094 (3)	0.3148 (5)	0.4387 (4)	0.0270 (14)	0.817 (4)
H3	0.6712	0.3196	0.4047	0.032*	0.817 (4)
S1'	0.7030 (3)	0.3001 (8)	0.4502 (6)	0.027*	0.183 (4)
C11	0.7226 (3)	0.4923 (5)	0.0199 (4)	0.0344 (13)	0.778 (4)
H11	0.7273	0.5338	−0.0300	0.041*	0.778 (4)
C12'	0.7283 (10)	0.478 (2)	0.0239 (13)	0.034*	0.222 (4)
H12'	0.7443	0.5110	−0.0261	0.041*	0.222 (4)
C12	0.6696 (3)	0.4975 (5)	0.0719 (3)	0.0329 (12)	0.778 (4)
H12	0.6334	0.5439	0.0634	0.039*	0.778 (4)
C11'	0.6679 (10)	0.4979 (18)	0.0575 (11)	0.033*	0.222 (4)
H11'	0.6370	0.5460	0.0339	0.039*	0.222 (4)
C13	0.6751 (3)	0.4259 (4)	0.1393 (4)	0.0283 (12)	0.778 (4)
H13	0.6422	0.4187	0.1812	0.034*	0.778 (4)
S2'	0.6554 (2)	0.4254 (6)	0.1468 (4)	0.028*	0.222 (4)
C19	0.5478 (2)	0.6236 (3)	0.5854 (3)	0.0439 (12)	0.960 (4)
H19	0.5209	0.6844	0.5761	0.053*	0.960 (4)
C20'	0.545 (2)	0.600 (5)	0.573 (6)	0.044*	0.040 (4)
H20'	0.5089	0.6463	0.5616	0.053*	0.040 (4)
C20	0.6113 (2)	0.6170 (3)	0.5612 (3)	0.0374 (10)	0.960 (4)
H20	0.6343	0.6723	0.5327	0.045*	0.960 (4)
C19'	0.607 (2)	0.621 (4)	0.549 (5)	0.037*	0.040 (4)
H19'	0.6205	0.6834	0.5193	0.045*	0.040 (4)
C21	0.6388 (2)	0.5186 (4)	0.5835 (3)	0.0277 (8)	0.960 (4)
H21	0.6828	0.5002	0.5711	0.033*	0.960 (4)
S3'	0.6590 (11)	0.521 (2)	0.580 (2)	0.028*	0.040 (4)
C29	0.5823 (3)	0.1198 (5)	0.9760 (5)	0.0365 (17)	0.665 (4)
H29	0.5894	0.0584	1.0101	0.044*	0.665 (4)
C30'	0.5976 (7)	0.1367 (11)	0.9825 (8)	0.037*	0.335 (4)
H30'	0.6171	0.0879	1.0210	0.044*	0.335 (4)
C30	0.5329 (3)	0.1272 (5)	0.9190 (5)	0.0352 (15)	0.665 (4)
H30	0.5029	0.0715	0.9063	0.042*	0.665 (4)
C29'	0.5429 (7)	0.1157 (11)	0.9377 (8)	0.035*	0.335 (4)
H29'	0.5193	0.0505	0.9407	0.042*	0.335 (4)
C31	0.5325 (5)	0.2282 (8)	0.8817 (8)	0.039 (2)	0.665 (4)
H31	0.4999	0.2503	0.8427	0.046*	0.665 (4)
S4'	0.5213 (3)	0.2209 (6)	0.8748 (4)	0.039*	0.335 (4)
F1	0.58969 (11)	0.6069 (2)	0.3032 (3)	0.0653 (9)	
F2	0.63964 (14)	0.7449 (2)	0.3477 (3)	0.0697 (9)	
F3	0.63082 (13)	0.7177 (3)	0.2175 (2)	0.0758 (11)	
F4	0.58607 (10)	0.10854 (18)	0.2800 (3)	0.0557 (7)	
F5	0.63759 (19)	0.0490 (4)	0.3863 (2)	0.1062 (16)	
F6	0.64439 (12)	−0.02908 (19)	0.2702 (3)	0.0724 (10)	
F7	0.4634 (2)	0.1221 (3)	0.5841 (4)	0.0691 (19)	0.637 (8)
F8	0.5383 (3)	0.0168 (6)	0.5542 (5)	0.118 (3)	0.637 (8)
F9	0.4967 (3)	0.0071 (5)	0.6696 (4)	0.088 (2)	0.637 (8)
F7'	0.4514 (3)	0.1011 (7)	0.6466 (7)	0.087 (3)	0.363 (8)
F8'	0.5011 (5)	0.0915 (7)	0.5354 (4)	0.071 (3)	0.363 (8)
F9'	0.5227 (3)	−0.0136 (4)	0.6325 (6)	0.050 (2)	0.363 (8)
F10	0.50376 (13)	0.7510 (2)	0.9026 (2)	0.0550 (7)	
F11	0.46071 (15)	0.6715 (3)	0.7974 (2)	0.0724 (9)	
F12	0.44353 (12)	0.6167 (2)	0.9230 (3)	0.0645 (8)	
O1	0.74885 (10)	0.63817 (17)	0.2411 (2)	0.0245 (5)	
O2	0.75368 (11)	0.10238 (17)	0.3191 (2)	0.0255 (5)	
O3	0.61131 (12)	0.11162 (18)	0.6867 (2)	0.0287 (5)	
O4	0.59885 (11)	0.62983 (18)	0.8317 (2)	0.0281 (5)	
N1	0.81933 (13)	0.4767 (2)	0.3145 (2)	0.0214 (5)	
H1N	0.8150 (17)	0.526 (2)	0.2758 (18)	0.026*	
N2	0.80754 (13)	0.2661 (2)	0.2295 (2)	0.0227 (5)	
H2N	0.8117 (18)	0.210 (2)	0.263 (2)	0.027*	
N3	0.66015 (13)	0.3092 (2)	0.6906 (2)	0.0206 (5)	
H3N	0.6634 (19)	0.2397 (11)	0.699 (2)	0.025*	
N4	0.65239 (12)	0.4362 (2)	0.8506 (2)	0.0188 (5)	
H4N	0.6551 (18)	0.5034 (12)	0.836 (2)	0.023*	
C4	0.76321 (17)	0.3778 (2)	0.4254 (2)	0.0273 (7)	
C5	0.76608 (15)	0.4627 (2)	0.3615 (2)	0.0218 (6)	
C6	0.71094 (15)	0.5277 (3)	0.3520 (2)	0.0244 (6)	
H6	0.6749	0.5164	0.3887	0.029*	
C7	0.70615 (15)	0.6077 (2)	0.2919 (2)	0.0223 (6)	
C8	0.64054 (16)	0.6680 (3)	0.2888 (3)	0.0308 (7)	
C9	0.87353 (15)	0.4019 (2)	0.3033 (2)	0.0231 (6)	
H9A	0.8760	0.3536	0.3527	0.028*	
H9B	0.9152	0.4420	0.2998	0.028*	
C10	0.86425 (15)	0.3358 (3)	0.2227 (2)	0.0230 (6)	
H10A	0.8585	0.3844	0.1739	0.028*	
H10B	0.9038	0.2922	0.2125	0.028*	
C14	0.73144 (15)	0.3671 (2)	0.1403 (2)	0.0243 (6)	
C15	0.74753 (16)	0.2808 (2)	0.2002 (2)	0.0218 (6)	
C16	0.69566 (16)	0.2139 (3)	0.2243 (2)	0.0250 (6)	
H16	0.6540	0.2252	0.1994	0.030*	
C17	0.70247 (15)	0.1316 (3)	0.2830 (3)	0.0238 (6)	
C18	0.64150 (18)	0.0659 (3)	0.3048 (3)	0.0358 (8)	
C22	0.59719 (15)	0.4511 (2)	0.6247 (2)	0.0248 (6)	
C23	0.60659 (15)	0.3393 (2)	0.6502 (2)	0.0213 (6)	
C24	0.55877 (15)	0.2635 (3)	0.6291 (2)	0.0256 (6)	
H24	0.5203	0.2865	0.6008	0.031*	
C25	0.56605 (16)	0.1554 (3)	0.6486 (3)	0.0274 (7)	
C26	0.51242 (19)	0.0802 (3)	0.6161 (3)	0.0467 (11)	
C27	0.71201 (15)	0.3739 (2)	0.7275 (2)	0.0200 (6)	
H27A	0.7079	0.4487	0.7080	0.024*	
H27B	0.7552	0.3465	0.7092	0.024*	
C28	0.70705 (15)	0.3695 (2)	0.8237 (2)	0.0194 (6)	
H28A	0.7000	0.2950	0.8424	0.023*	
H28B	0.7482	0.3958	0.8494	0.023*	
C32	0.58421 (15)	0.2942 (2)	0.9067 (2)	0.0237 (6)	
C33	0.59499 (15)	0.4057 (2)	0.8802 (2)	0.0195 (6)	
C34	0.54391 (16)	0.4786 (3)	0.8887 (2)	0.0243 (6)	
H34	0.5039	0.4554	0.9132	0.029*	
C35	0.54967 (15)	0.5854 (3)	0.8622 (2)	0.0238 (6)	
C36	0.48897 (16)	0.6564 (3)	0.8712 (3)	0.0331 (8)	

### Atomic displacement parameters (Å^2^)

**Table:**

	*U*^11^	*U*^22^	*U*^33^	*U*^12^	*U*^13^	*U*^23^
S1	0.0389 (6)	0.0307 (6)	0.0202 (5)	0.0018 (4)	−0.0086 (4)	0.0065 (4)
S2	0.0444 (8)	0.0286 (6)	0.0196 (6)	−0.0034 (5)	−0.0023 (4)	0.0044 (4)
S3	0.0345 (5)	0.0312 (5)	0.0388 (5)	0.0102 (4)	−0.0073 (4)	−0.0019 (4)
S4	0.0385 (8)	0.0278 (7)	0.0347 (8)	−0.0062 (6)	−0.0076 (6)	0.0157 (6)
C1	0.052 (3)	0.0195 (19)	0.0158 (17)	0.0066 (19)	0.0069 (18)	0.0055 (14)
C2	0.034 (2)	0.0191 (18)	0.029 (2)	0.0017 (16)	0.0095 (18)	0.0034 (16)
C3	0.028 (2)	0.009 (2)	0.044 (3)	−0.0054 (16)	0.021 (2)	0.0011 (19)
C11	0.064 (4)	0.017 (3)	0.022 (2)	−0.009 (2)	−0.019 (2)	0.0030 (16)
C12	0.052 (3)	0.020 (2)	0.027 (3)	−0.0032 (19)	−0.022 (2)	0.0014 (19)
C13	0.037 (3)	0.024 (2)	0.025 (2)	−0.009 (3)	−0.003 (3)	−0.0018 (17)
C19	0.071 (3)	0.0204 (19)	0.040 (2)	0.0077 (19)	−0.025 (2)	−0.0027 (17)
C20	0.066 (3)	0.0229 (18)	0.0238 (19)	−0.0040 (18)	−0.0130 (18)	0.0034 (14)
C21	0.030 (2)	0.031 (2)	0.0221 (16)	−0.004 (2)	−0.002 (2)	−0.0017 (13)
C29	0.040 (4)	0.018 (3)	0.051 (4)	−0.007 (2)	0.011 (3)	0.019 (3)
C30	0.031 (3)	0.024 (3)	0.050 (4)	−0.010 (2)	0.001 (3)	0.004 (3)
C31	0.039 (5)	0.018 (3)	0.058 (5)	0.002 (3)	−0.012 (3)	0.008 (3)
F1	0.0253 (11)	0.0377 (14)	0.133 (3)	0.0008 (10)	0.0128 (14)	0.0210 (15)
F2	0.0597 (17)	0.0509 (16)	0.098 (2)	0.0229 (14)	−0.0003 (17)	−0.0277 (16)
F3	0.0454 (15)	0.115 (3)	0.0672 (18)	0.0397 (16)	0.0135 (13)	0.0494 (18)
F4	0.0242 (10)	0.0285 (12)	0.114 (2)	0.0029 (8)	0.0065 (13)	0.0132 (13)
F5	0.083 (2)	0.180 (4)	0.0556 (18)	−0.080 (3)	0.0037 (16)	0.037 (2)
F6	0.0357 (12)	0.0205 (12)	0.161 (3)	−0.0042 (9)	0.0048 (17)	−0.0111 (15)
F7	0.041 (2)	0.052 (2)	0.115 (4)	−0.0208 (19)	−0.053 (3)	0.029 (3)
F8	0.105 (4)	0.119 (5)	0.131 (5)	−0.029 (4)	−0.014 (3)	−0.083 (4)
F9	0.057 (3)	0.096 (4)	0.110 (4)	−0.052 (3)	−0.032 (3)	0.056 (3)
F7'	0.055 (4)	0.083 (5)	0.122 (6)	−0.022 (4)	0.018 (4)	−0.015 (4)
F8'	0.097 (5)	0.064 (5)	0.052 (4)	−0.038 (4)	−0.033 (4)	0.009 (3)
F9'	0.039 (4)	0.029 (3)	0.081 (5)	−0.009 (3)	−0.030 (3)	0.000 (3)
F10	0.0432 (13)	0.0328 (13)	0.089 (2)	0.0075 (10)	0.0100 (13)	−0.0235 (13)
F11	0.0778 (19)	0.081 (2)	0.0579 (16)	0.0553 (18)	−0.0245 (15)	−0.0128 (16)
F12	0.0347 (13)	0.0500 (15)	0.109 (2)	0.0131 (11)	0.0295 (15)	0.0152 (16)
O1	0.0239 (11)	0.0206 (11)	0.0291 (11)	−0.0008 (8)	0.0029 (9)	0.0048 (9)
O2	0.0278 (11)	0.0187 (11)	0.0299 (11)	0.0014 (9)	0.0003 (9)	0.0029 (9)
O3	0.0288 (12)	0.0240 (12)	0.0332 (12)	−0.0046 (9)	−0.0100 (10)	0.0038 (10)
O4	0.0264 (11)	0.0207 (11)	0.0373 (12)	−0.0001 (9)	0.0027 (10)	0.0046 (9)
N1	0.0242 (12)	0.0166 (12)	0.0233 (13)	0.0012 (10)	−0.0013 (10)	0.0016 (10)
N2	0.0296 (14)	0.0164 (12)	0.0220 (12)	−0.0012 (10)	0.0006 (11)	0.0006 (10)
N3	0.0246 (13)	0.0159 (12)	0.0213 (12)	−0.0027 (10)	−0.0020 (10)	0.0002 (10)
N4	0.0206 (11)	0.0141 (12)	0.0219 (12)	−0.0004 (10)	0.0001 (10)	0.0007 (9)
C4	0.0436 (19)	0.0223 (16)	0.0159 (14)	0.0046 (14)	0.0039 (13)	0.0016 (12)
C5	0.0270 (15)	0.0193 (15)	0.0190 (13)	−0.0017 (12)	−0.0008 (12)	−0.0005 (11)
C6	0.0280 (15)	0.0221 (15)	0.0230 (15)	−0.0001 (13)	0.0070 (12)	0.0017 (12)
C7	0.0225 (14)	0.0201 (15)	0.0242 (14)	−0.0023 (11)	0.0001 (12)	−0.0018 (12)
C8	0.0259 (16)	0.0235 (16)	0.0429 (19)	0.0013 (13)	0.0060 (15)	0.0049 (15)
C9	0.0209 (14)	0.0219 (15)	0.0264 (15)	0.0024 (12)	−0.0025 (12)	0.0037 (12)
C10	0.0234 (14)	0.0211 (15)	0.0245 (14)	0.0008 (12)	0.0021 (12)	0.0037 (12)
C14	0.0333 (16)	0.0196 (14)	0.0199 (14)	−0.0010 (12)	−0.0051 (13)	−0.0011 (12)
C15	0.0295 (16)	0.0189 (15)	0.0170 (13)	0.0006 (12)	−0.0016 (12)	−0.0032 (11)
C16	0.0263 (15)	0.0236 (16)	0.0251 (15)	0.0018 (12)	−0.0044 (13)	−0.0019 (12)
C17	0.0258 (15)	0.0177 (14)	0.0279 (15)	−0.0011 (11)	0.0029 (13)	−0.0011 (12)
C18	0.0297 (17)	0.0284 (18)	0.049 (2)	−0.0022 (14)	−0.0015 (16)	0.0045 (16)
C22	0.0311 (16)	0.0238 (15)	0.0194 (14)	0.0014 (13)	−0.0072 (12)	0.0007 (12)
C23	0.0242 (14)	0.0215 (14)	0.0184 (13)	−0.0006 (12)	0.0000 (11)	0.0001 (11)
C24	0.0248 (15)	0.0259 (16)	0.0262 (15)	−0.0018 (12)	−0.0076 (13)	0.0019 (12)
C25	0.0268 (15)	0.0299 (17)	0.0255 (15)	−0.0051 (13)	−0.0056 (13)	0.0022 (13)
C26	0.048 (2)	0.032 (2)	0.060 (3)	−0.0172 (18)	−0.023 (2)	0.0123 (19)
C27	0.0207 (14)	0.0177 (14)	0.0217 (14)	−0.0024 (11)	−0.0018 (12)	−0.0019 (11)
C28	0.0207 (14)	0.0157 (13)	0.0219 (13)	0.0017 (11)	−0.0008 (11)	0.0002 (11)
C32	0.0250 (15)	0.0206 (15)	0.0256 (15)	−0.0001 (12)	0.0029 (12)	0.0034 (12)
C33	0.0215 (14)	0.0208 (14)	0.0163 (13)	−0.0045 (11)	−0.0008 (11)	−0.0004 (11)
C34	0.0207 (14)	0.0241 (16)	0.0279 (15)	−0.0020 (12)	0.0013 (12)	0.0044 (12)
C35	0.0223 (14)	0.0227 (16)	0.0264 (15)	0.0017 (12)	−0.0008 (12)	0.0002 (12)
C36	0.0260 (16)	0.0271 (18)	0.046 (2)	0.0035 (14)	0.0005 (15)	−0.0016 (15)

### Geometric parameters (Å, °)

**Table:**

S1—C4	1.682 (3)	S4'—C32	1.659 (6)
S1—C1	1.704 (4)	F1—C8	1.311 (4)
C3'—C4	1.408 (10)	F2—C8	1.340 (5)
C3'—C2'	1.438 (11)	F3—C8	1.304 (4)
C3'—H3'	0.9500	F4—C18	1.313 (4)
S2—C11	1.710 (5)	F5—C18	1.310 (5)
S2—C14	1.731 (3)	F6—C18	1.310 (5)
C13'—C14	1.351 (10)	F7—C26	1.240 (5)
C13'—C12'	1.421 (11)	F8—C26	1.368 (6)
C13'—H13'	0.9500	F9—C26	1.288 (5)
S3—C19	1.701 (4)	F7'—C26	1.363 (6)
S3—C22	1.716 (3)	F8'—C26	1.306 (6)
C21'—C22	1.357 (12)	F9'—C26	1.222 (6)
C21'—C20'	1.420 (12)	F10—C36	1.318 (4)
C21'—H21'	0.9500	F11—C36	1.317 (5)
S4—C32	1.697 (3)	F12—C36	1.336 (5)
S4—C29	1.707 (5)	O1—C7	1.248 (4)
C31'—C32	1.387 (9)	O2—C17	1.248 (4)
C31'—C30'	1.422 (11)	O3—C25	1.233 (4)
C31'—H31'	0.9500	O4—C35	1.247 (4)
C1—C2	1.367 (6)	N1—C5	1.331 (4)
C1—H1	0.9500	N1—C9	1.462 (4)
C2'—C1'	1.352 (11)	N1—H1N	0.875 (12)
C2'—H2'	0.9500	N2—C15	1.325 (4)
C2—C3	1.404 (6)	N2—C10	1.455 (4)
C2—H2	0.9500	N2—H2N	0.878 (12)
C1'—S1'	1.705 (11)	N3—C23	1.323 (4)
C1'—H1'	0.9500	N3—C27	1.457 (4)
C3—C4	1.371 (5)	N3—H3N	0.882 (12)
C3—H3	0.9500	N4—C33	1.321 (4)
S1'—C4	1.618 (7)	N4—C28	1.459 (4)
C11—C12	1.362 (6)	N4—H4N	0.874 (12)
C11—H11	0.9500	C4—C5	1.468 (4)
C12'—C11'	1.368 (11)	C5—C6	1.399 (4)
C12'—H12'	0.9500	C6—C7	1.385 (4)
C12—C13	1.398 (6)	C6—H6	0.9500
C12—H12	0.9500	C7—C8	1.541 (4)
C11'—S2'	1.701 (11)	C9—C10	1.533 (4)
C11'—H11'	0.9500	C9—H9A	0.9900
C13—C14	1.367 (6)	C9—H9B	0.9900
C13—H13	0.9500	C10—H10A	0.9900
S2'—C14	1.722 (6)	C10—H10B	0.9900
C19—C20	1.358 (6)	C14—C15	1.476 (4)
C19—H19	0.9500	C15—C16	1.405 (4)
C20'—C19'	1.360 (12)	C16—C17	1.394 (5)
C20'—H20'	0.9500	C16—H16	0.9500
C20—C21	1.400 (5)	C17—C18	1.533 (5)
C20—H20	0.9500	C22—C23	1.470 (4)
C19'—S3'	1.709 (12)	C23—C24	1.402 (4)
C19'—H19'	0.9500	C24—C25	1.397 (5)
C21—C22	1.364 (5)	C24—H24	0.9500
C21—H21	0.9500	C25—C26	1.534 (5)
S3'—C22	1.697 (11)	C27—C28	1.528 (4)
C29—C30	1.357 (7)	C27—H27A	0.9900
C29—H29	0.9500	C27—H27B	0.9900
C30'—C29'	1.351 (11)	C28—H28A	0.9900
C30'—H30'	0.9500	C28—H28B	0.9900
C30—C31	1.396 (9)	C32—C33	1.474 (4)
C30—H30	0.9500	C33—C34	1.394 (4)
C29'—S4'	1.708 (10)	C34—C35	1.406 (4)
C29'—H29'	0.9500	C34—H34	0.9500
C31—C32	1.399 (7)	C35—C36	1.534 (4)
C31—H31	0.9500		
			
C4—S1—C1	92.9 (2)	C5—C6—H6	118.3
C4—C3'—C2'	111.3 (7)	O1—C7—C6	128.0 (3)
C4—C3'—H3'	124.3	O1—C7—C8	116.0 (3)
C2'—C3'—H3'	124.3	C6—C7—C8	116.0 (3)
C11—S2—C14	92.0 (2)	F3—C8—F1	107.9 (3)
C14—C13'—C12'	112.7 (7)	F3—C8—F2	104.9 (3)
C14—C13'—H13'	123.6	F1—C8—F2	106.7 (3)
C12'—C13'—H13'	123.6	F3—C8—C7	113.2 (3)
C19—S3—C22	92.3 (2)	F1—C8—C7	113.6 (3)
C22—C21'—C20'	112.0 (9)	F2—C8—C7	110.0 (3)
C22—C21'—H21'	124.0	N1—C9—C10	110.7 (2)
C20'—C21'—H21'	124.0	N1—C9—H9A	109.5
C32—S4—C29	91.8 (2)	C10—C9—H9A	109.5
C30'—C31'—H31'	124.2	N1—C9—H9B	109.5
C2—C1—S1	112.0 (3)	C10—C9—H9B	109.5
C2—C1—H1	124.0	H9A—C9—H9B	108.1
S1—C1—H1	124.0	N2—C10—C9	111.1 (2)
C1'—C2'—C3'	110.2 (8)	N2—C10—H10A	109.4
C1'—C2'—H2'	124.9	C9—C10—H10A	109.4
C3'—C2'—H2'	124.9	N2—C10—H10B	109.4
C1—C2—C3	110.1 (4)	C9—C10—H10B	109.4
C1—C2—H2	124.9	H10A—C10—H10B	108.0
C3—C2—H2	124.9	C13'—C14—C15	130.1 (7)
C2'—C1'—S1'	112.8 (10)	C13—C14—C15	126.1 (3)
C2'—C1'—H1'	123.6	C13'—C14—S2'	111.2 (7)
S1'—C1'—H1'	123.6	C15—C14—S2'	118.2 (3)
C4—C3—C2	114.9 (4)	C13—C14—S2	109.4 (3)
C4—C3—H3	122.5	C15—C14—S2	124.3 (2)
C2—C3—H3	122.5	N2—C15—C16	121.5 (3)
C4—S1'—C1'	93.3 (6)	N2—C15—C14	122.2 (3)
C12—C11—S2	112.1 (4)	C16—C15—C14	116.3 (3)
C12—C11—H11	123.9	C17—C16—C15	123.1 (3)
S2—C11—H11	123.9	C17—C16—H16	118.5
C11'—C12'—C13'	112.5 (9)	C15—C16—H16	118.5
C11'—C12'—H12'	123.7	O2—C17—C16	127.3 (3)
C13'—C12'—H12'	123.7	O2—C17—C18	115.0 (3)
C11—C12—C13	111.6 (4)	C16—C17—C18	117.7 (3)
C11—C12—H12	124.2	F6—C18—F5	105.6 (4)
C13—C12—H12	124.2	F6—C18—F4	106.4 (3)
C12'—C11'—S2'	111.3 (10)	F5—C18—F4	107.9 (4)
C12'—C11'—H11'	124.4	F6—C18—C17	110.9 (3)
S2'—C11'—H11'	124.4	F5—C18—C17	111.0 (3)
C14—C13—C12	115.0 (4)	F4—C18—C17	114.6 (3)
C14—C13—H13	122.5	C21—C22—C23	129.7 (3)
C12—C13—H13	122.5	C23—C22—S3'	120.8 (11)
C11'—S2'—C14	92.3 (6)	C21—C22—S3	109.5 (3)
C20—C19—S3	112.1 (3)	C23—C22—S3	120.3 (2)
C20—C19—H19	124.0	N3—C23—C24	120.0 (3)
S3—C19—H19	124.0	N3—C23—C22	120.8 (3)
C19'—C20'—C21'	112.6 (9)	C24—C23—C22	119.2 (3)
C19'—C20'—H20'	123.7	C25—C24—C23	121.9 (3)
C21'—C20'—H20'	123.7	C25—C24—H24	119.0
C19—C20—C21	111.4 (4)	C23—C24—H24	119.0
C19—C20—H20	124.3	O3—C25—C24	128.2 (3)
C21—C20—H20	124.3	O3—C25—C26	115.3 (3)
C20'—C19'—S3'	111.2 (11)	C24—C25—C26	116.4 (3)
C20'—C19'—H19'	124.4	F7—C26—F9	111.5 (4)
S3'—C19'—H19'	124.4	F9'—C26—F8'	110.0 (5)
C22—C21—C20	114.6 (4)	F9'—C26—F7'	105.6 (5)
C22—C21—H21	122.7	F7—C26—F8	105.4 (4)
C20—C21—H21	122.7	F9—C26—F8	98.9 (4)
C30—C29—S4	113.6 (4)	F9'—C26—C25	113.3 (3)
C30—C29—H29	123.2	F7—C26—C25	117.1 (3)
S4—C29—H29	123.2	F9—C26—C25	113.3 (3)
C29'—C30'—C31'	111.9 (8)	F8'—C26—C25	112.8 (4)
C29'—C30'—H30'	124.0	F7'—C26—C25	114.7 (4)
C31'—C30'—H30'	124.0	F8—C26—C25	108.6 (4)
C29—C30—C31	110.3 (5)	N3—C27—C28	109.4 (2)
C29—C30—H30	124.8	N3—C27—H27A	109.8
C31—C30—H30	124.8	C28—C27—H27A	109.8
C30'—C29'—S4'	111.7 (10)	N3—C27—H27B	109.8
C30'—C29'—H29'	124.1	C28—C27—H27B	109.8
S4'—C29'—H29'	124.1	H27A—C27—H27B	108.2
C30—C31—C32	114.2 (5)	N4—C28—C27	108.7 (2)
C30—C31—H31	122.9	N4—C28—H28A	109.9
C32—C31—H31	122.9	C27—C28—H28A	109.9
C32—S4'—C29'	92.8 (6)	N4—C28—H28B	109.9
C5—N1—C9	127.1 (3)	C27—C28—H28B	109.9
C5—N1—H1N	114 (2)	H28A—C28—H28B	108.3
C9—N1—H1N	116 (2)	C31'—C32—C33	124.4 (6)
C15—N2—C10	129.0 (3)	C31—C32—C33	126.2 (4)
C15—N2—H2N	114 (2)	C33—C32—S4'	123.6 (3)
C10—N2—H2N	116 (2)	C31—C32—S4	109.9 (4)
C23—N3—C27	129.7 (3)	C33—C32—S4	123.6 (2)
C23—N3—H3N	114 (3)	S4'—C32—S4	112.5 (3)
C27—N3—H3N	116 (3)	N4—C33—C34	120.7 (3)
C33—N4—C28	128.2 (3)	N4—C33—C32	120.6 (3)
C33—N4—H4N	115 (2)	C34—C33—C32	118.7 (3)
C28—N4—H4N	115 (3)	C33—C34—C35	122.1 (3)
C3—C4—C5	123.7 (4)	C33—C34—H34	119.0
C3'—C4—C5	118.2 (7)	C35—C34—H34	119.0
C3'—C4—S1'	112.4 (7)	O4—C35—C34	127.4 (3)
C5—C4—S1'	129.3 (4)	O4—C35—C36	115.5 (3)
C3—C4—S1	109.8 (3)	C34—C35—C36	117.1 (3)
C5—C4—S1	126.0 (3)	F10—C36—F11	107.8 (3)
N1—C5—C6	121.5 (3)	F10—C36—F12	105.3 (3)
N1—C5—C4	120.9 (3)	F11—C36—F12	107.0 (3)
C6—C5—C4	117.6 (3)	F10—C36—C35	111.7 (3)
C7—C6—C5	123.5 (3)	F11—C36—C35	110.8 (3)
C7—C6—H6	118.3	F12—C36—C35	113.8 (3)
			
C4—S1—C1—C2	0.1 (3)	C14—C15—C16—C17	−177.3 (3)
C4—C3'—C2'—C1'	0.2 (5)	C15—C16—C17—O2	−3.3 (5)
S1—C1—C2—C3	−2.5 (5)	C15—C16—C17—C18	178.9 (3)
C3'—C2'—C1'—S1'	−0.1 (2)	O2—C17—C18—F6	−72.0 (4)
C1—C2—C3—C4	4.4 (7)	C16—C17—C18—F6	106.1 (4)
C2'—C1'—S1'—C4	−0.1 (2)	O2—C17—C18—F5	45.0 (5)
C14—S2—C11—C12	1.4 (4)	C16—C17—C18—F5	−136.9 (4)
C14—C13'—C12'—C11'	−0.1 (5)	O2—C17—C18—F4	167.5 (3)
S2—C11—C12—C13	−1.4 (5)	C16—C17—C18—F4	−14.3 (5)
C13'—C12'—C11'—S2'	0.0 (2)	C20'—C21'—C22—C21	−1.4 (16)
C11—C12—C13—C14	0.7 (6)	C20'—C21'—C22—C23	−166 (3)
C12'—C11'—S2'—C14	0.02 (18)	C20'—C21'—C22—S3'	0.0 (6)
C22—S3—C19—C20	0.7 (3)	C20—C21—C22—C23	173.5 (3)
C22—C21'—C20'—C19'	0.0 (5)	C20—C21—C22—S3'	−163 (9)
S3—C19—C20—C21	−0.3 (4)	C20—C21—C22—S3	0.9 (4)
C21'—C20'—C19'—S3'	0.0 (2)	C19'—S3'—C22—C21'	0.0 (4)
C19—C20—C21—C22	−0.4 (5)	C19—S3—C22—C21	−0.9 (3)
C20'—C19'—S3'—C22	−0.01 (19)	C19—S3—C22—C23	−174.3 (3)
C32—S4—C29—C30	1.2 (5)	C19—S3—C22—S3'	2.1 (15)
C32—C31'—C30'—C29'	0.0 (5)	C27—N3—C23—C24	−171.8 (3)
S4—C29—C30—C31	−3.1 (8)	C27—N3—C23—C22	9.9 (5)
C31'—C30'—C29'—S4'	0.0 (2)	C21'—C22—C23—N3	−150 (3)
C29—C30—C31—C32	4.0 (12)	C21—C22—C23—N3	48.7 (5)
C30'—C29'—S4'—C32	0.00 (19)	S3'—C22—C23—N3	44.2 (15)
C2—C3—C4—C3'	2.3 (11)	S3—C22—C23—N3	−139.5 (3)
C2—C3—C4—C5	−176.2 (4)	C21'—C22—C23—C24	31 (3)
C2—C3—C4—S1'	−12 (6)	C21—C22—C23—C24	−129.6 (4)
C2—C3—C4—S1	−4.3 (6)	S3'—C22—C23—C24	−134.1 (15)
C2'—C3'—C4—C3	−1.9 (9)	S3—C22—C23—C24	42.2 (4)
C2'—C3'—C4—C5	176.7 (5)	N3—C23—C24—C25	−0.9 (5)
C2'—C3'—C4—S1'	−0.2 (6)	C22—C23—C24—C25	177.4 (3)
C1'—S1'—C4—C3'	0.2 (4)	C23—C24—C25—O3	2.1 (6)
C1'—S1'—C4—C5	−176.4 (5)	C23—C24—C25—C26	−175.6 (3)
C1'—S1'—C4—S1	−6.0 (5)	O3—C25—C26—F9'	−0.5 (7)
C1—S1—C4—C3	2.3 (4)	C24—C25—C26—F9'	177.5 (6)
C1—S1—C4—C5	174.0 (3)	O3—C25—C26—F7	173.8 (5)
C1—S1—C4—S1'	3.2 (4)	C24—C25—C26—F7	−8.2 (6)
C9—N1—C5—C6	−164.6 (3)	O3—C25—C26—F9	41.8 (6)
C9—N1—C5—C4	15.3 (5)	C24—C25—C26—F9	−140.1 (5)
C3—C4—C5—N1	−138.4 (5)	O3—C25—C26—F8'	−126.4 (6)
C3'—C4—C5—N1	43.1 (7)	C24—C25—C26—F8'	51.7 (7)
S1'—C4—C5—N1	−140.6 (5)	O3—C25—C26—F7'	120.8 (7)
S1—C4—C5—N1	51.0 (4)	C24—C25—C26—F7'	−61.2 (7)
C3—C4—C5—C6	41.4 (6)	O3—C25—C26—F8	−67.0 (6)
C3'—C4—C5—C6	−137.1 (7)	C24—C25—C26—F8	111.0 (5)
S1'—C4—C5—C6	39.3 (6)	C23—N3—C27—C28	108.1 (3)
S1—C4—C5—C6	−129.1 (3)	C33—N4—C28—C27	107.6 (3)
N1—C5—C6—C7	2.6 (5)	N3—C27—C28—N4	−75.1 (3)
C4—C5—C6—C7	−177.3 (3)	C30'—C31'—C32—C31	−0.5 (10)
C5—C6—C7—O1	−3.7 (5)	C30'—C31'—C32—C33	175.9 (6)
C5—C6—C7—C8	177.7 (3)	C30'—C31'—C32—S4'	0.0 (6)
O1—C7—C8—F3	20.9 (5)	C30'—C31'—C32—S4	112 (20)
C6—C7—C8—F3	−160.3 (3)	C30—C31—C32—C31'	−1.4 (14)
O1—C7—C8—F1	144.5 (3)	C30—C31—C32—C33	−177.7 (6)
C6—C7—C8—F1	−36.8 (4)	C30—C31—C32—S4'	−171 (20)
O1—C7—C8—F2	−96.0 (4)	C30—C31—C32—S4	−3.1 (12)
C6—C7—C8—F2	82.7 (4)	C29'—S4'—C32—C31'	0.0 (4)
C5—N1—C9—C10	96.2 (4)	C29'—S4'—C32—C33	−175.9 (5)
C15—N2—C10—C9	98.3 (4)	C29'—S4'—C32—S4	−1.7 (5)
N1—C9—C10—N2	−65.9 (3)	C29—S4—C32—C31	1.0 (7)
C12'—C13'—C14—C13	−4.0 (7)	C29—S4—C32—C33	175.8 (4)
C12'—C13'—C14—C15	172.0 (10)	C29—S4—C32—S4'	1.6 (4)
C12'—C13'—C14—S2'	0.1 (6)	C28—N4—C33—C34	−168.3 (3)
C12—C13—C14—C13'	1.0 (11)	C28—N4—C33—C32	13.1 (4)
C12—C13—C14—C15	−175.2 (4)	C31'—C32—C33—N4	54.7 (7)
C12—C13—C14—S2	0.4 (6)	C31—C32—C33—N4	−129.5 (8)
C11'—S2'—C14—C13'	−0.1 (4)	S4'—C32—C33—N4	−129.9 (4)
C11'—S2'—C14—C15	−173.0 (9)	S4—C32—C33—N4	56.6 (4)
C11'—S2'—C14—S2	−1.2 (9)	C31'—C32—C33—C34	−123.9 (6)
C11—S2—C14—C13	−1.0 (4)	C31—C32—C33—C34	51.9 (9)
C11—S2—C14—C15	174.7 (3)	S4'—C32—C33—C34	51.5 (5)
C11—S2—C14—S2'	3.4 (4)	S4—C32—C33—C34	−122.1 (3)
C10—N2—C15—C16	−169.2 (3)	N4—C33—C34—C35	3.6 (5)
C10—N2—C15—C14	11.3 (5)	C32—C33—C34—C35	−177.8 (3)
C13'—C14—C15—N2	41.9 (11)	C33—C34—C35—O4	−2.6 (5)
C13—C14—C15—N2	−142.9 (4)	C33—C34—C35—C36	177.2 (3)
S2'—C14—C15—N2	−146.6 (4)	O4—C35—C36—F10	−44.5 (4)
S2—C14—C15—N2	42.2 (4)	C34—C35—C36—F10	135.7 (3)
C13'—C14—C15—C16	−137.6 (11)	O4—C35—C36—F11	75.8 (4)
C13—C14—C15—C16	37.6 (5)	C34—C35—C36—F11	−104.0 (4)
S2'—C14—C15—C16	33.9 (4)	O4—C35—C36—F12	−163.5 (3)
S2—C14—C15—C16	−137.3 (3)	C34—C35—C36—F12	16.6 (5)
N2—C15—C16—C17	3.2 (5)		

### Hydrogen-bond geometry (Å, °)

**Table:**

*D*—H···*A*	*D*—H	H···*A*	*D*···*A*	*D*—H···*A*
N1—H1n···O1	0.88 (1)	2.03 (3)	2.741 (3)	138 (3)
N2—H2n···O2	0.88 (1)	2.01 (3)	2.726 (3)	138 (3)
N3—H3n···O3	0.88 (1)	1.93 (3)	2.668 (3)	140 (3)
N4—H4n···O4	0.87 (1)	1.96 (3)	2.677 (3)	139 (3)
